# Metabolic profiles of regulatory T cells and their adaptations to the tumor microenvironment: implications for antitumor immunity

**DOI:** 10.1186/s13045-022-01322-3

**Published:** 2022-08-10

**Authors:** Yuheng Yan, Lan Huang, Yiming Liu, Ming Yi, Qian Chu, Dechao Jiao, Kongming Wu

**Affiliations:** 1 Department of Oncology, Tongji Hospital of Tongji Medical College, Huazhong University of Science and Technology, Wuhan, 430030 China; 2 Biotherapy Center, The First Affiliated Hospital of Zhengzhou University, Zhengzhou, 450052 China; 3 Department of Interventional Radiology, The First Affiliated Hospital of Zhengzhou University, Zhengzhou, 450052 China

**Keywords:** Regulatory T cell, Tumor microenvironment, Glycolysis, Oxidative phosphorylation, Fatty acid oxidation, Fatty acid synthesis, Amino acid metabolism, Immunotherapy

## Abstract

Characterized by the expression of the critical transcription factor forkhead box protein P3, regulatory T (Treg) cells are an essential part of the immune system, with a dual effect on the pathogenesis of autoimmune diseases and cancer. Targeting Tregs to reestablish the proinflammatory and immunogenic tumor microenvironment (TME) is an increasingly attractive strategy for cancer treatment and has been emphasized in recent years. However, attempts have been significantly hindered by the subsequent autoimmunity after Treg ablation owing to systemic loss of their suppressive capacity. Cellular metabolic reprogramming is acknowledged as a hallmark of cancer, and emerging evidence suggests that elucidating the underlying mechanisms of how intratumoral Tregs acquire metabolic fitness and superior immunosuppression in the TME may contribute to clinical benefits. In this review, we discuss the common and distinct metabolic profiles of Tregs in peripheral tissues and the TME, as well as the differences between Tregs and other conventional T cells in their metabolic preferences. By focusing on the critical roles of different metabolic programs, such as glycolysis, oxidative phosphorylation, fatty acid oxidation, fatty acid synthesis, and amino acid metabolism, as well as their essential regulators in modulating Treg proliferation, migration, and function, we hope to provide new insights into Treg cell-targeted antitumor immunotherapies.

## Introduction

Malignant tumors are characterized by unlimited proliferation, invasion, and metastasis, which are attributed not only to diverse genomic alterations but also, as evidenced by increasing studies, to the support of the surrounding tumor microenvironment (TME) [1–4]. Tumor-infiltrating lymphocytes (TILs), considered critical components of the extracellular milieu, play dual roles in modulating cancer progression [5, 6]. On the one hand, tumor cells are under immunosurveillance in the presence of various proinflammatory cells, such as CD8^+^ cytotoxic T cells, CD4^+^ type 1 helper T (Th1) cells, and natural killer cells. However, these cells usually develop into exhausted functional states and fail to elicit sufficient antitumor immunity [7–10]. On the other hand, the recruitment of excessive immunosuppressive cells, including tolerogenic dendritic cells, myeloid-derived suppressive cells (MDSCs), tumor-associated macrophages, Th2 cells, and regulatory T (Treg) cells, as well as the secretion of a plethora of cytokines, supports the establishment of an immunosuppressive TME, thus promoting tumor immune evasion [11–17]. Among them, Treg cells, which represent the master regulatory cells and participate in the maintenance of immune homeostasis, are regarded as the chief obstacle to antitumor immunity [18].

The expression of chemokine receptors, particularly chemokine (C–C motif) receptor 4 (CCR4), CCR7, CCR8, C–X–C chemokine receptor type 4 (CXCR4), and CXCR5 [19, 20], contributes to the abundant accumulation of Tregs in the TME, where they are metabolically reprogrammed and functionally adapted to the low-nutrient, high-lactate environment. It is widely acknowledged that cellular metabolic reprogramming is a hallmark of cancer [1, 21]. Furthermore, manipulation of cellular metabolism on the viability and function of both cancer cells and immune cells has aroused growing concern in the past decade, and considerable efforts have been made in checkpoint blockade immunotherapy and adoptive cellular treatment [22, 23]. Intriguingly, in March 2021, three research findings were simultaneously published in *Nature*, exploring the metabolic determinants for Treg survival and suppressive function in the TME [24–26]. These studies indicate that further understanding the metabolic requirements of intratumoral Tregs may provide an opportunity to intervene in the immunosuppressive TME and enhance antitumor immunity. In this review, we elaborate on the characteristics of Treg metabolism and metabolic determinants involved in regulating Treg proliferation, migration, and suppressive capacity. We also discuss the mechanisms underlying Treg functional fitness in the TME, hoping to find potential approaches to reestablish proinflammatory circumstances by perturbing these metabolic programs, ultimately enhancing the efficacy of antitumor immunity.

## A brief introduction of Tregs: discovery, phenotypes, and functions

The induction and maintenance of immunological tolerance, which can prevent excessive immune response to self-antigens and induce immunological homeostasis, have always been central issues in the research field of autoimmune diseases, allergies, and organ transplantation for decades [27]. Early in the 1970s, Gershon et al. proposed that thymus-derived “suppressor” cells exhibit immunologically tolerant effects, and this function is sustained during transference from antigen-tolerant mice to thymectomized and lethally irradiated secondary hosts [28, 29]. Subsequently, several works further explored the role of this new T lymphocyte subset on inflammation and autoimmune diseases such as azobenzenersonate-induced granulomas, experimental allergic encephalomyelitis, and delayed-type hypersensitivity, as well as cancers [30–34]. However, progress was greatly hampered due to the lack of knowledge on their specific surface biomarkers for detection and classification. Moreover, the mechanisms of activation and suppression could not be determined. It was not until the late 1990s that the scientific community’s attention was drawn back to the investigation of T suppressor cells, as significant breakthroughs were made by Sakaguchi et al. on establishing a new concept of “regulatory” T or Treg cells, together with demonstrating the expression of IL-2 receptor α-chain (CD25) on this CD5^high^CD45RB^low^ CD4^+^ T cell subpopulation [35]. However, the expression pattern of CD4^+^ CD25^+^ was not specific to regulatory T cells, and it was difficult to distinguish this autoimmune-preventive cell subset from memory, effector, or activated T cells. Furthermore, Sakaguchi et al. subsequently discovered that the forkhead transcription factor forkhead box P3 (Foxp3), which is encoded and specifically expressed in CD4^+^CD25^+^ Treg cells, played a dominant role in Treg developmental differentiation and suppressive function [36]. The mutation or genetic deletion of Foxp3 caused a severe autoimmune disease known as IPEX syndrome [36]. As Foxp3 could serve as a more specific marker for discriminating Treg cells and other T cells [37, 38], CD4^+^CD25^+^Foxp3^+^ is referred to as a classical combined marker of regulatory T cells, influencing later studies [39].

### Classifications and phenotypes of Tregs

**Fig. 1:**
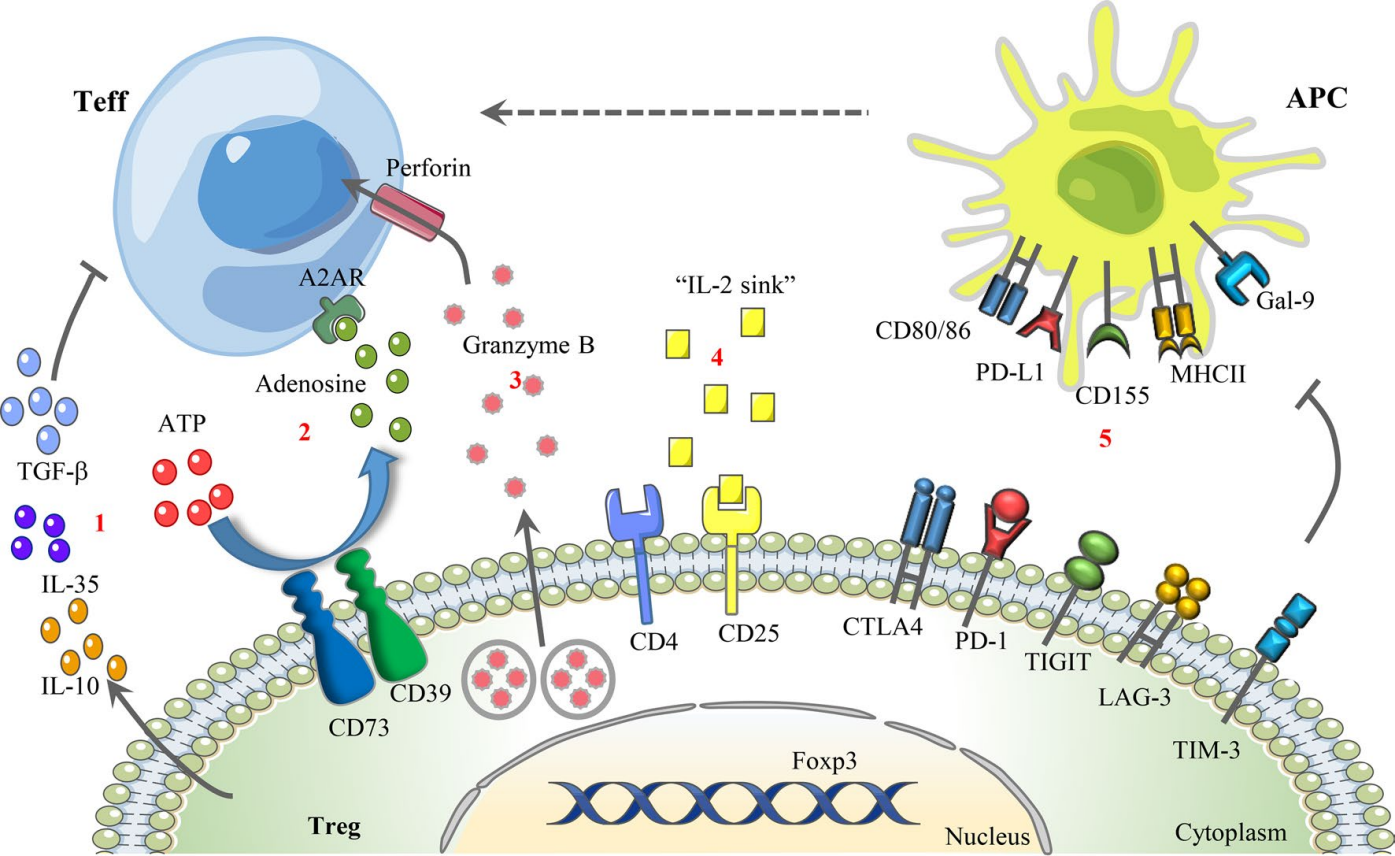
Underlying mechanisms of Treg-mediated immunosuppression**.** From left to right: **1** Secretion of immunosuppressive cytokines, such as TGF-β, IL-10, and IL-35. **2** Suppression of Teff activity via adenosine-A2AR signaling. **3** Granzyme B/perforin-dependent cytolysis of target cells via direct cell–cell contact. **4** “IL-2 sink.” **5** Inhibition of antigen-presenting cell maturation by selectively expressing cell surface suppressor receptors, such as CTLA-4, PD-1, TIGIT, LAG-3, and TIM-3, the receptors of which are CD80/86, PD-L1, CD155, MHC-II, and Gal-9. *A2AR* A2A receptor, *APC* antigen-presenting cell, *TIGIT* T cell immunoreceptor with immunoglobulin and ITIM domains, *LAG-3* lymphocyte activation gene 3, *TIM-3* T cell immunoglobulin mucin-domain-containing-3, *Gal-9* Galectin-9

Based on the generation site, Tregs are divided into two major subsets, thymic Tregs (tTregs), also named natural Tregs (nTregs), and peripheral Tregs (pTregs)/induced Tregs (iTregs). To standardize the nomenclature of Tregs and simplify future research, several scholars jointly wrote an article to Nature Immunology, suggesting that Tregs be uniformly categorized as tTregs and pTregs [40]. tTregs are initially generated from the thymus, where they are stimulated by self-antigens presented by thymus epithelial cells. Once activated, they are transported to the periphery, exhibiting suppressive activities against self-antigens [41, 42]. Differently, pTregs play essential roles in preventing autoimmune responses against foreign antigens and are converted from naïve or conventional T cells (Tcons) in peripheral tissues under certain conditions, including stimulation from cytokines such as tumor transformation factor (TGF)-β and IL-2, as well as T cell antigen receptor (TCR) signal transduction [41, 42]. Additionally, tTregs show higher stability than pTregs owing to the stable expression of Foxp3 induced by DNA hypomethylation in the gene conserved noncoding sequences 2 (CNS2) locus and the expression of transcripts, such as signal transducer and activator of transcription 5 (STAT5) and cyclic AMP response element-binding protein [43]. However, both tTregs and nTregs infiltrate the TME, exhibiting immunosuppressive functions [44, 45], and nTregs, which highly express Helios, are the main component of tumor-infiltrating Tregs (TI-Tregs) [46]. Moreover, human Tregs are functionally and phenotypically heterogeneous and can be classified into three subpopulations based on their phenotypes [47, 48]: Fraction I (Fr. I), Foxp3^low^CD25^low^CD45RA^+^ cells, referred to as naïve or resting Tregs; Fr. II, Foxp3^high^CD25^high^CD45RA^−^ cells, referred to as effector Tregs (eTregs); and Fr. III, FOXP3^low^CD25^low^CD45RA^−^ cells, referred to as non-Tregs, which have no suppressive function but can secrete proinflammatory cytokines. In the TME, self-antigens released by dying cancer cells induce the conversion of naïve Tregs to eTregs via TCR stimulation [42, 45]. eTregs express diverse cell surface biomarkers, such as cytotoxic T lymphocyte antigen 4 (CTLA-4), programmed cell death protein-1 (PD-1), T cell immunoreceptor with immunoglobulin and ITIM domains (TIGIT), lymphocyte activation gene 3 (LAG-3), and T cell immunoglobulin mucin-domain-containing-3 (TIM3), thus acquiring higher proliferation and suppression capacity (Fig. 1) [42, 45].

### Functional properties of Tregs in cancer and underlying mechanisms

Accumulating evidence demonstrates that Tregs are not only actively engaged in suppressing abnormal immune responses against self-antigens but also play dominant roles in impairing antitumor responses and promoting tumorigenesis [42]. The high infiltration rate of Tregs in the TME correlates with the poor prognosis of patients in diverse malignancies, such as non-small cell lung cancer [49], ovarian cancer [50, 51], glioblastoma (GBM) [52], and pancreatic ductal adenocarcinoma [53, 54]. However, some studies based on other certain malignancies, such as head and neck squamous cell cancer [55], follicular lymphoma [56], colorectal cancer [57], and gastric cancer [58], confirmed the positive correlation between Treg infiltration and the increased survival rate of cancer patients. These contradictory results may be explained by the differences in tumor etiology, tumor stages, and the heterogeneity of Tregs in phenotype and function [42].

The underlying mechanisms of Treg-mediated immunosuppression can be briefly categorized into the following five parts (Fig. 1): (1) secretion of immunosuppressive cytokines such as TGF-β, IL-10, and IL-35; (2) granzyme B/perforin- or Fas-FasL-dependent cytolysis of target cells via direct cell–cell contact; (3) inhibition of antigen-presenting cell maturation by expressing cell surface suppressor receptors, such as CTLA-4, PD-1, TIGIT, LAG-3, and TIM-3; (4) act as an “IL-2 sink” and result in IL-2 exhaustion in the milieu; and (5) regulation of effector T cell (Teff) function through metabolic changes, such as promoting adenosine accumulation and competing for nutrients [59–62]. Cellular metabolism drives different biological processes of T cells, and the reprogramming of T cell metabolism in the immune-suppressive TME has drawn significant attention in recent years [63, 64]. The involvement of Tregs in modulating Teff DNA damage and senescence has been extensively proven, partially due to the superior metabolic competitiveness of Tregs in the TME compared with Teffs [60, 61]. Additionally, it was confirmed that Tregs derived from ovarian and gastric cancer tissues are capable of hydrolyzing ATP into adenosine through the ectonucleotidases CD39 and CD73, and the secretion of adenosine suppresses the immune response by binding to the A2A receptor expressed on CD8^+^ T cells (Fig. 1) [62, 65]. Likewise, in right-sided colorectal cancer, the abnormal activation of phospholipase a2-IVa/arachidonic signaling induced the generation of CD39^+^γδ Treg cells, which mediated immunosuppressive effects via the secretion of IL-17A and adenosine [66].

Elucidating the mechanisms of Treg suppression will facilitate the development of Treg-targeted antitumor immunotherapy, and the participation of metabolic alterations of cancer cells and immune cells in cancer pathogenesis and progression should not be neglected.

## Metabolic determinants for Treg proliferation, migration, and function

Metabolic and energetic pathways are vital for cell survival and function. Similar to other cells, immune cells often switch their metabolic programs to meet increased energy demand and biosynthesis upon activation [67]. During the past 10 years, the intricate linkage between inflammation and metabolism has been highlighted. A new field of research called “immunometabolism”, which has taken on the meaning of “metabolic programming and reprogramming of immune cell generation, development, and function”, provides new insights into the treatment of inflammatory diseases and cancer [67].

### Specific characteristics of Treg metabolism and its key regulators

**Fig. 2:**
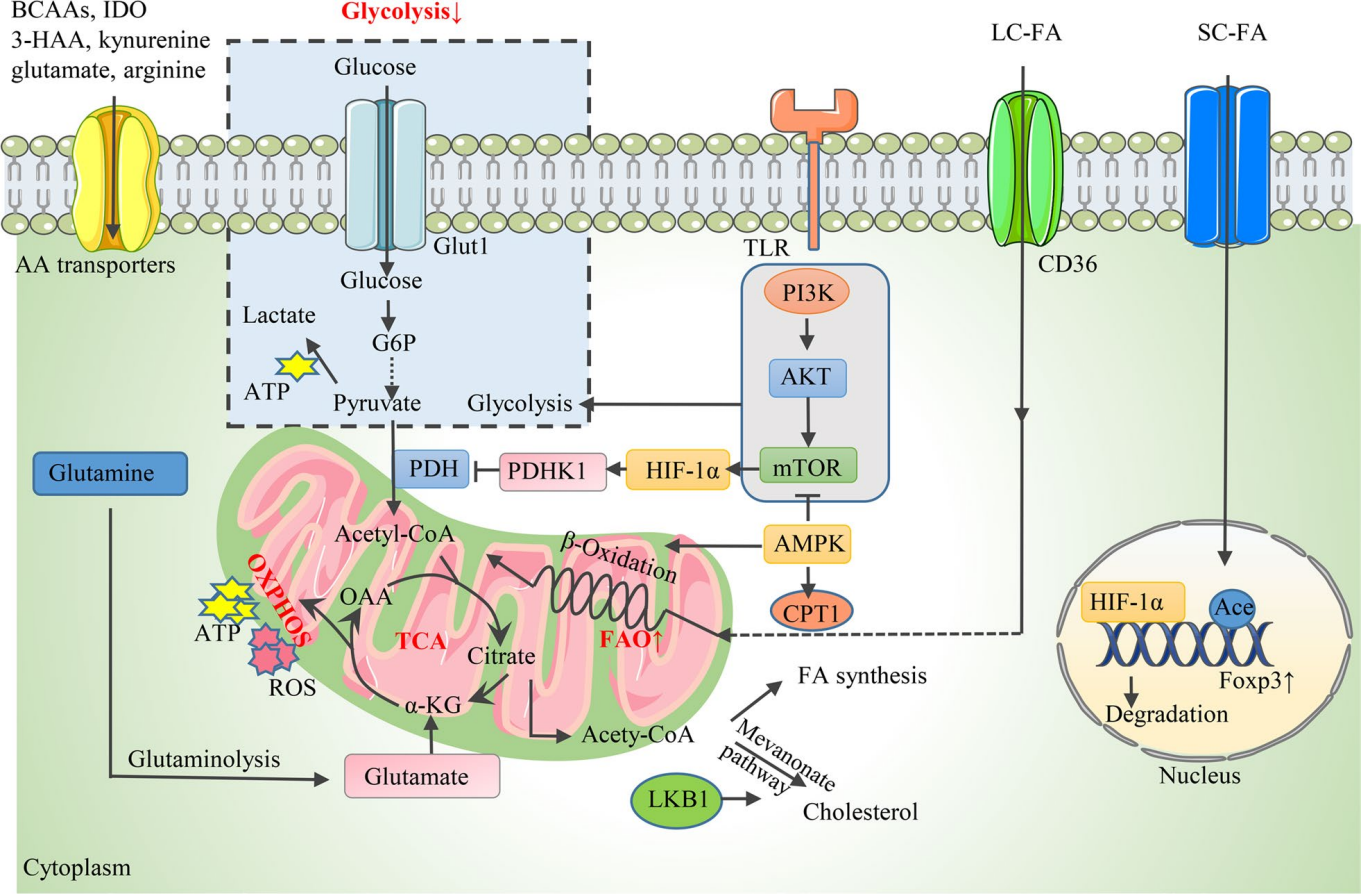
Metabolic determinants for Treg proliferation. During proliferation, Tregs exhibit decreased glycolysis but increased FAO. **1** The glycolytic rate is regulated by the PI3K-AKT-mTOR axis along with its upstream and downstream molecules, including AMPK and HIF-1α. AMPK inhibits glycolysis by downregulating mTOR activity. HIF-1α is activated by mTOR and modulates Treg proliferation through different mechanisms, including binding to Foxp3 and causing its degradation, as well as promoting the curbing of PDHK1 on PDH, resulting in a glycolysis shift and OXPHOS blockade. **2** AMPK activates CPT1 and drives mitochondrial FAO. LKB1, the upstream regulator of AMPK, engages the mevalonate pathway to produce cholesterol. SC-FA promotes Foxp3 expression by mediating histone H3 Ace in the promoter region of Foxp3. **3** BCAAs, IDO, tryptophan metabolites (3-HAA, kynurenine), glutamate, and arginine support Treg proliferation. *AMPK* AMP-activated protein kinase, *HIF-1*α hypoxia-inducible factor 1 α, *PDHK1* pyruvate dehydrogenase kinase 1, *L/S C-FA* long/short-chain fatty acid, *CPT1* carnitine palmitoyltransferase-1, *LKB1* liver kinase B1, *Ace* acetylation, *BCAA* branched-chain amino acids, *IDO* indoleamine 2,3-dioxygenase, *3-HAA* 3-hydroxyanthranillic acid (The process of mitochondria respiration was adapted from Fig. 1 in [139])

As a specific immunosuppressive T cell subset, Tregs are highly heterogeneous with other T cells in metabolic features. Generally, proinflammatory cells, such as Teffs and M1 macrophages, utilize glycolysis as a rapid energy production method to fuel their increased energy demand during expansion and inflammatory function [68]. Differently, Tregs, as well as memory CD8^+^ T cells, predominantly depend on oxidative phosphorylation (OXPHOS) and fatty acid oxidation (FAO), which provide more efficient energy sources [68]. The preference of Tregs for lipid metabolism has been extensively studied in visceral adipose tissue (VAT), correlating with adipose tissue inflammation and pathogenesis of metabolic abnormalities such as type 2 diabetes, obesity, and fatty liver disease [69]. VAT-resident Tregs, distinguishable from lymphoid organ Tregs by their distinct transcriptomes, T cell receptor repertoires, and chemokine/chemokine receptor expression profiles, are orchestrated by peroxisome proliferator-activated receptor (PPAR)-γ, a type of nuclear transcription factor [69]. As the master regulator of adipocyte differentiation, PPAR-γ is expressed in VAT Tregs and is pivotal for peroxisomal-mediated β-oxidation of FAO [69]. Additionally, PPAR-γ-dependent upregulation of the cell surface lipid scavenger, CD36, was also observed [69]. Likewise, Tregs were also specifically recruited to the intestinal mucosa, where abundant dietary fiber metabolite short-chain FAs (e.g., butyrate, acetate, and propionate) were produced [70]. A positive correlation was found between short-chain FA levels and Treg number, and the effects of butyrate and propionate on Tregs were mediated by elevated histone H3 acetylation in the promoter region of Foxp3 (Fig. 2) [71, 72]. In addition to PPAR-γ, it was demonstrated that leptin receptors were expressed on VAT Tregs and other Tregs. The high leptin level in the adipose tissue of obese mice inhibited Treg generation, leading to local inflammation and insulin resistance [73]. The mechanism underlying leptin-induced suppression was that leptin decreased STAT3 and ERK1/2 phosphorylation and upregulated the cell cycle inhibitor P27^kip1^, thus increasing Treg apoptosis. The activation of mTOR is also involved in this process [74].

Metabolic regulators orchestrate Treg metabolism through different mechanisms. The glycolytic rate of Tregs is tightly regulated by diverse metabolic signals, the most significant of which is the phosphoinositide 3-kinase (PI3K)-Akt [also defined as protein kinase B]-mammalian target of rapamycin (mTOR) signaling network. Accumulating evidence suggests that the PI3K-Akt-mTOR pathway enhances the glycolytic rate of Treg cells and significantly influences their differentiation and functional stability [75–78]. Notably, mTOR is composed of two complexes [mTOR complex 1 (mTORC1) and mTORC2], and mTORC2 hyperactivation followed by specific mTORC1 deletion promotes glycolytic metabolism and thus inhibits Treg activity [79–81]. The activity of the PI3K-Akt-mTOR pathway is regulated by several critical upstream and downstream molecules, such as AMP-activated protein kinase (AMPK), phosphatase and tensin homolog (PTEN), and hypoxia-inducible factor 1 α (HIF-1α). For example, AMPK is an essential regulator of mammalian energy metabolism stimulated to induce a metabolic switch of the organism to catabolism by the increased ratio of AMP/ATP in the condition of cellular nutrient shortage or physiological stress [82]. Notably, AMPK was demonstrated to be an active regulator of both glycolysis and FAO in Tregs [83, 84].

Moreover, acting as potential substrates to fuel the tricarboxylic acid (TCA) cycle, amino acids are engaged in multiple metabolic processes. Amino acid availability and metabolism regulates immune homeostasis and responses. Amino acid transporters, branched-chain amino acids (BCAAs), glutamate, glutamine, glutathione (GSH), serine, and the catabolism of tryptophan and arginine were proved to modulate Treg generation and function.

Tregs plastically manipulate glycolysis, OXPHOS, FAO, and amino acid metabolism to meet energy demand and adapt to environmental changes. However, the interconnections between these processes and the underlying mechanisms are controversial and remain obscure.

### Proliferative Tregs primarily rely on FAO

It has been largely documented that Tregs disfavor glycolysis for proliferation [68, 85], consistent with the observation that glucose deprivation or treatment with the glycolysis inhibitor 2-deoxyglucose (2-DG) promotes Treg generation [86]. The PI3K-Akt-mTOR pathway is positively involved in regulating the glycolytic rate of Tregs and inhibits proliferation (Fig. 2). Activated Akt was also confirmed to interfere with the nuclear localization of Foxo and downregulate the transcription of Foxp3, thus impairing the differentiation of both nTregs and iTregs [77]. Treatment with PI3K, Akt, or mTOR inhibitors increased the number of activated CD4^+^ T cells and Foxp3 expression [78]. AMPK acts as the upstream inhibitor of mTOR activity, the inhibition of mTOR via the AMPK activator metformin contributed to decreased glycolysis in T cells, thus promoting Treg generation with the suppression of Th1 and Th17 cells [83, 84]. In addition, the oxygen-sensitive transcription factor HIF-1α was the critical downstream target of mTOR, and HIF-1α inhibition induced Treg differentiation while decreasing the Th17 number by impairing glycolytic activity [86]. Mechanistically, HIF-1α could bind to Foxp3 and cause its degradation [87]. Moreover, pyruvate dehydrogenase kinase 1 (PDHK1) was activated by HIF-1α and curbed the activity of PDH, which catalyzed the conversion of pyruvate to acetyl-CoA to enter the TCA cycle. Dysfunction of PDH resulted in a glycolysis shift and OXPHOS blockade in Tregs, thereby leading to a decreased Treg/Th17 ratio [88, 89]. Also of note, critical glucose transporter 1 (Glut1), which is essential for the CD4^+^ Teff cell-intrinsic metabolic program to drive cellular expansion and induce inflammation in diseases such as colitis and graft-versus-host disease, is expressed at low levels on Tregs and is not required for Treg activation [90].

However, several studies also confirmed that proliferating Tregs utilized glycolysis to fuel additional energy demand by enhancing mTOR activity [91–93]. The activation of PI3K-Akt-mTORC1 signaling through Toll-like receptor (TLR) signals increased Glut1-mediated glucose uptake and the glycolysis rate of Tregs, thus promoting Treg proliferation at the expense of their suppressive function [75]. Additionally, studies proved that HIF-1α was essential for Treg abundance and function under hypoxia by increasing the expression of Foxp3 [94, 95]. The contradictory results might be attributed to the following reasons: (1) differences in the proliferative and functional status of Tregs; (2) different experimental settings, such as in vivo or in vitro culture systems, human or mouse models; and (3) distinct nutrient and cytokine compositions within the extracellular environment.

Lipid oxidation is the primary metabolic resource for Treg generation. Key enzymes and transporters orchestrating FA metabolism, including carnitine palmitoyltransferase-1 (CPT1), AMPK, liver kinase B1 (LKB1), and CD36, play essential roles in this process (Fig. 2) [70, 96, 97]. CPT1 is the key rate-limiting enzyme of FAO, catalyzing the conjugation of long-chain fatty acids and carnitine to produce the precursor of acetyl-CoA, acyl-CoA, which is the substrate for β-oxidation [85]. Pharmacological inhibition of CPT1 through etomoxir restrained the proliferation of Tregs [85]. However, it seemed contrasting that the genetic deletion of CPT1 failed to affect the production and function of both murine tTregs and pTregs, indicating that etomoxir influenced T cell activity via different targets in addition to CPT1 [98, 99]. As an essential metabolic sensor, AMPK was found to activate CPT1 and drive mitochondrial FAO for ATP generation while inhibiting the anabolism of FA [68]. Notably, LKB1, the upstream regulator of AMPK, participates in Treg generation and function through different mechanisms. It can not only regulate FAO and OXPHOS in an AMPK-independent manner but also engage the mevalonate pathway to produce lipids cholesterol and the isoprenoid geranylgeranyl pyrophosphate as extra energy resources [100–102].

Amino acid availability and metabolism play crucial roles in modulating Treg generation (Fig. 2). BCAAs, which include leucine, isoleucine, and valine, were proven to be required for Treg proliferation in a BCAA transporter Slc3a2-dependent manner [103]. Treatment with BCAA-reduced diets or depletion of Slc3a2 impaired the in vivo expansion of Treg cells in murine models [103]. Similarly, the cystine/glutamate antiporter solute carrier Slc7a11, the expression of which was regulated by nuclear factor erythroid 2-related factor 2 (NRF2), has recently been discovered to elevate Treg proliferation capacity [104]. Another study showed that the increased extracellular glutamate level induced by the overexpression of the glutamate/cystine antiporter Slc7a11/xCT on glioma cells promoted Treg proliferation in vitro, consistent with the result obtained with exogenous glutamate supplementation in Treg-only cultures [105]. Similarly, glutamate is converted by glutamine, the deprivation of which shifts the balance between Th1 and Treg cells toward that of a Foxp3^+^ Treg phenotype even in the presence of cytokines with Th1-inducing effects [106]. In addition, tryptophan and arginine are two of the essential amino acids regulating the immune response [107]. The catabolism of tryptophan is predominantly mediated by the rate-limiting enzyme indoleamine 2,3-dioxygenase (IDO), and increasing evidence shows that IDO and tryptophan metabolites in the IDO-catalyzed kynurenine pathway, including kynurenine and 3-hydroxyanthranillic acid (3-HAA), promote Treg proliferation [108, 109]. Mechanistically, 3-HAA interacted with TGF-β-secreting dendritic cells (DCs) to increase the Treg number in vitro [108]. A recent study indicated that the absence of arginine resulted in a reduced Treg number in a murine model, suggesting that arginine is pivotal for Treg production [110].

In summary, Tregs utilize FA as primary metabolic substrates for proliferation, whereas glycolysis is dispensable for Treg differentiation, and metabolism also plays an important role in regulating Treg generation and immune homeostasis.

### Glycolysis is crucial for Treg migration

**Fig. 3:**
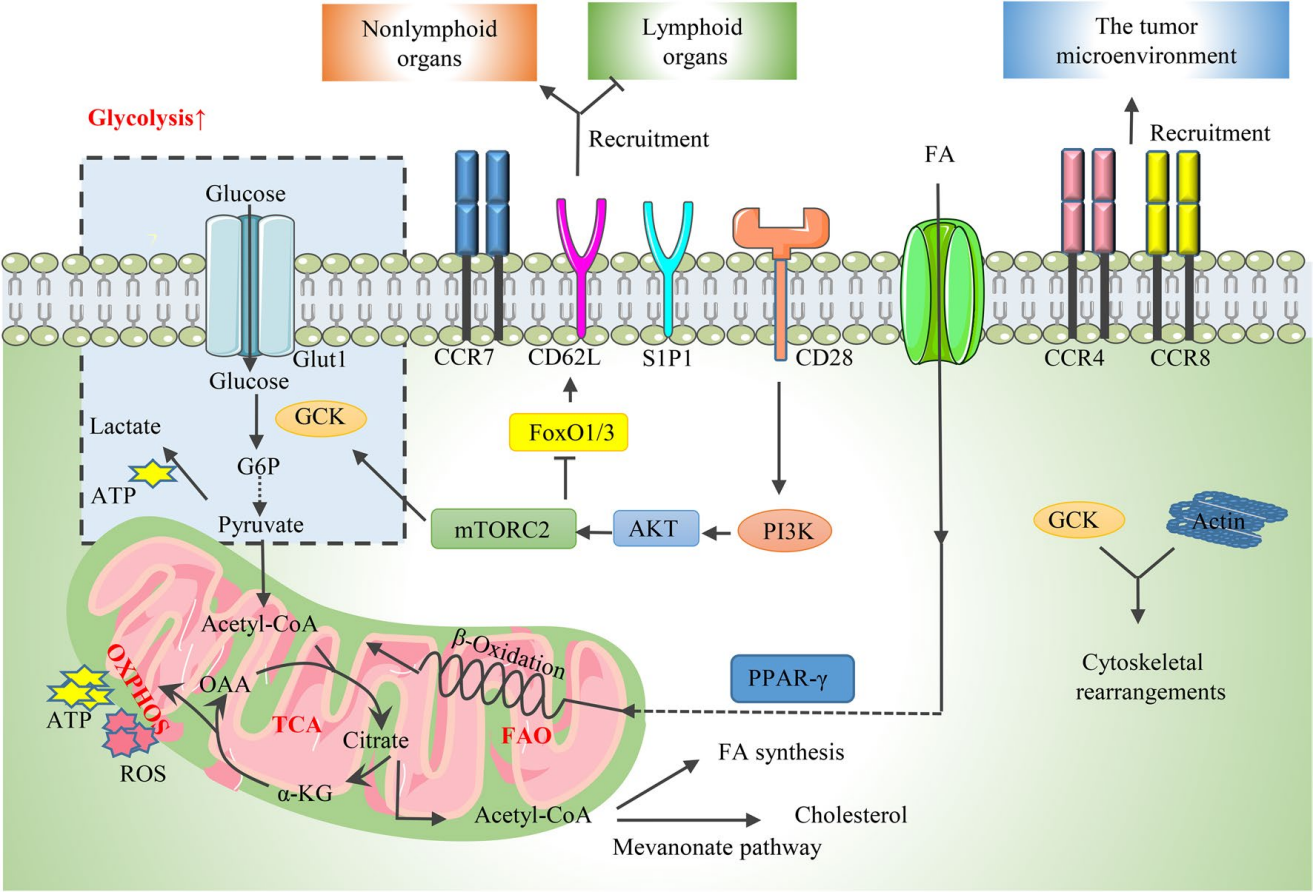
Metabolic determinants for Treg migration. Tregs exhibit increased glycolysis during migration. **1** CCR4 and CCR8 contribute to Treg abundance in the TME. **2** The PI3K-AKT-mTORC2 axis promotes glycolysis by activating GCK, which also interacts with actin to enhance cytoskeletal rearrangements during migration. Additionally, the PI3K-Akt-mTORC2 pathway inhibits CCR7, CD62L, and S1P1 by suppressing the FoxO1 and FoxO3 transcription factors, thereby strongly influencing Treg migration in a way that favors Treg access to peripheral nonlymphoid organs while impairing their recirculation in lymphoid organs. **3** PPAR-γ plays an important role in driving the recruitment of Treg cells to sites of inflammation. *GCK* glucokinase, *S1P1* sphingosine-1-phosphate receptor 1, *FoxO1/3* Forkhead Box O1/3, *PPAR-γ* peroxisome proliferator-activated receptor-γ

The accumulation of Tregs in lymphoid and nonlymphoid tissues, such as the skin, liver, and TME, is orchestrated by the expression of diverse chemokine receptors, adhesion molecules, cytokines, and costimulatory and coinhibitory receptors, such as CD28 and CTLA-4 [41, 42]. It was reported that the upregulation of chemokine receptors, mainly CCR4 and CCR8, contributed to Treg abundance in the TME (Fig. 3) [41]. Although poorly researched, metabolic signaling pathways manipulating energy production are pivotal for Treg motility. Glycolysis, a rapid method for ATP production, is considered the key energy resource for supplying the migratory bioenergetic demands of Tregs [111]. The enhanced glycolytic flux is stimulated by CD28 signal-induced activation of the PI3K-Akt pathway, which unexpectedly mediates the expression of the enzyme glucokinase (GCK) by targeting downstream rapamycin-insensitive mTORC2, not mTORC1 (Fig. 3) [111]. Moreover, GCK interacts with actin to enhance cytoskeletal rearrangements during migration (Fig. 3) [111]. Additionally, the lack of the PI3K-Akt-mTORC2-GCK pathway reduced the migration of Tregs to skin allografts without affecting their suppressive function, together with the observation that human Tregs carrying a mutant GCK regulatory protein gene (resulting in increased GCK activity) showed decreased numbers but enhanced migration [111]. All these results indicate that glycolysis is vital for migration, impairs proliferation, and is not required for the suppressive function of Treg cells. Furthermore, the activation of the PI3K-Akt-mTORC2 pathway was investigated to inhibit CD62L, CCR7, and sphingosine-1-phosphate receptor 1 through suppressing forkhead Box O1 (FoxO1) and the FoxO3 transcription factor, thereby strongly influencing Treg migration in a way that favors Treg access to peripheral nonlymphoid organs while impairing their recirculation in lymphoid organs (Fig. 3) [93, 112]. Since the participation of PI3K-p110δ in promoting Treg migration has been extensively studied, PI3K p110δ antagonists, such as idelalisib, have been broadly applied as immune modulators to treat hematologic malignancies [113].

In recent years, accumulating evidence has demonstrated that, apart from glycolysis, lipid metabolism is also involved in the regulation of Treg migratory activity [114]. PPAR-γ, considered a critical regulator of adipocyte differentiation and β-oxidation of FAO, drives the recruitment of Treg cells to inflammation sites, thus blocking atherosclerosis progression (Fig. 3) [114]. Correspondingly, it was also reported that PPAR-γ served as a marker for the migration of splenic PPAR^lo^ Tregs, which contained precursors of multiple tissue-Treg compartments, to VAT, as well as nonlymphoid tissues, such as skin and liver [115]. Likewise, diet-induced dyslipidemia stimulated the PPAR-δ signal, which promoted the migration of splenic Tregs to inflamed tissues but not lymph nodes by promoting FAO [116]. Inhibition of mTORC1 was also observed in this study, indicating that FAO may compensate for the decreased glycolysis with large ATP production [116]. Notably, IDO is enriched in the TME, contributing to tryptophan exhaustion and Treg recruitment in the TME [117]. These results imply that amino acid metabolism is also involved in the regulation of Treg motility, but the mechanisms remain unclear.

It can be concluded that glycolysis serves as the critical energetic resource for Treg migration, and additional studies about the modulation of lipid and AA metabolism on Treg motility are needed.

### The suppressive function of Tregs is supported by mitochondrial metabolism

**Fig. 4:**
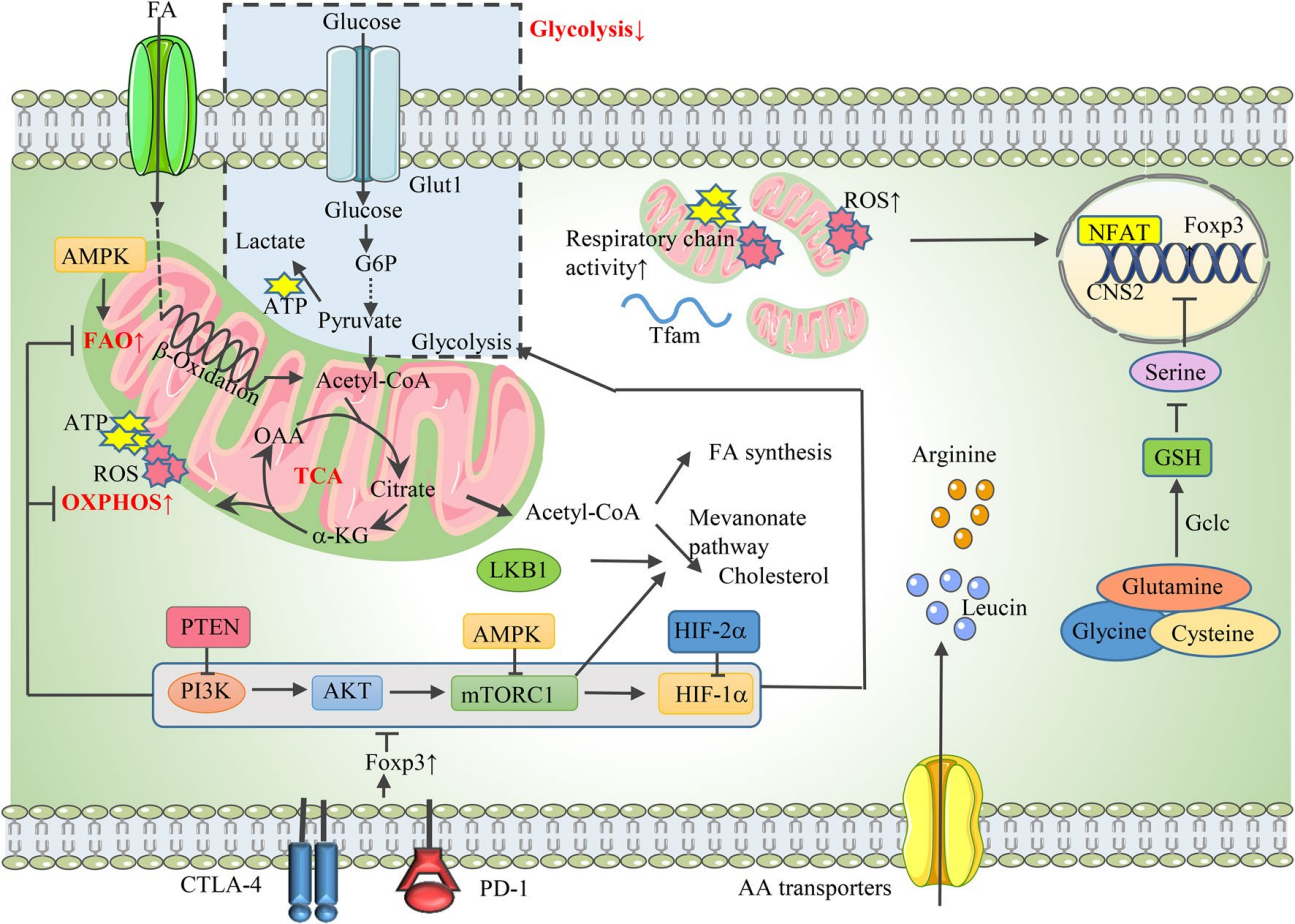
Tregs exhibit decreased glycolysis but increased FAO and OXPHOS in functional status. **1** The PI3K-Akt-mTOR-HIF-1α pathway negatively influences Treg function by promoting glycolysis at the cost of decreased OXPHOS and FAO, and this axis is inhibited by CTLA-4- and PD-1-stimulated Foxp3 expression. PTEN and AMPK are the upstream inhibitors of PI3K and mTORC1, respectively, promoting Treg functional stability. HIF-2α relieves the suppression of HIF-1α on Treg function. **2** Tregs show high mitochondrial mass and excessive ROS production, which promotes Foxp3 expression by activating the binding of NFAT on the CNS2 enhancer of Foxp3. Additionally, Tfam and the mitochondrial respiratory chain are essential for the maintenance of Treg suppressive capacity. **3** The increased FAO is stimulated by the high expression of AMPK. FAS, especially the mevalonate pathway, which is activated by raptor/mTORC1 signaling and LKB1, influences Treg function. **4** Amino acids, especially arginine and leucine, drive effector Treg function. Synthesized by the enzyme Gclc in the presence of glutamine, glycine, and cysteine, GSH enhances Foxp3 expression and stimulates Treg suppressive capacity by suppressing serine. *PTEN* phosphatase and tensin homolog, *ROS* reactive oxygen species, *NFAT* nuclear factor of activated T cells, *CNS2* conserved noncoding sequences 2, *Tfam* transcription factor A, *FAS* fatty acid synthesis, *Gclc* glutamate cysteine ligase, *GSH* glutathione

The stable expression of Foxp3 and the maintenance of Treg stability are critical for their suppressive function [118]. The stimulation of suppressive molecules on Tregs, including CTLA-4 and PD-1, increased the expression of Foxp3, which directly inhibited the PI3K-Akt-mTORC1 axis and caused a decrease in glycolysis and increases in OXPHOS and FAO (Fig. 4) [75, 119]. It was acknowledged that glycolysis and Glut1-induced glucose uptake diminished Treg function while increasing their proportion [75]. The PI3K-Akt-mTOR pathway and its upstream and downstream molecules, such as AMPK, PTEN, and HIF-1α, are regarded as the key regulators of the glycolytic flux of Tregs and strongly influence their differentiation and function. Furthermore, the involvement of these molecules in regulating the OXPHOS-driven suppressive activity of Treg cells was also uncovered (Fig. 4). For example, the inhibition of mTOR-mediated glycolysis through AMPK stimulated OXPHOS and FAO [85], and HIF-1α promoted glycolysis-fueled migration of Treg cells at the cost of OXPHOS-supported suppressive function [120]. A recent study showed that HIF-2α was a potential inhibitor of HIF-1α activity, and knockout of HIF-2α in Treg cells relieved its suppression of HIF-1α and thereby impaired the Treg capacity to inhibit effector T-cell-induced colitis and airway allergic inflammation [121]. In addition, the phosphatase PTEN is expressed on Treg cells and physiologically restrains the PI3K/Akt pathway to sustain Treg functional stability and suppressive capacity [122, 123]. PTEN ablation in Tregs resulted in their conversion from immunosuppressive T cells into proinflammatory effector cells, also referred to as ex-Tregs [124]. Notably, high mitochondrial mass and excessive reactive oxygen species (ROS) production were observed in both nTregs and iTregs [125]. Produced during OXPHOS, ROS promotes Foxp3 expression by activating the nuclear factor of activated T cells in the nucleus, which then binds to the CNS2 enhancer of the Foxp3 gene and stimulates its expression [126]. Moreover, the mitochondrial respiratory chain and mitochondrial transcription factor A (Tfam), which are both essential determinants of mitochondrial respiration, were also confirmed to be indispensable for the maintenance of Treg suppressive capacity [127, 128]. The specific ablation of mitochondrial complex III in murine Treg cells caused the loss of Treg suppressive function with unchanged expansion, while the genetic deletion of Tfam in Tregs impaired both their proliferation and function by enhancing DNA methylation in the TSDR of the Foxp3 locus, culminating in inflammatory dysfunction and tumor rejection [128].

Lipid metabolism actively participates in regulating Treg function [129]. Tregs exhibited increased FAO, which is stimulated by the high AMPK expression and signals transduced by CTLA-4 and PD-1, thus sustaining the ability to maintain tissue homeostasis (Fig. 4) [75, 85]. Additionally, Treg cells are regulated by fatty acid synthesis (FAS), especially the mevalonate pathway, which was activated by raptor/mTORC1 signaling and LKB1, and consequently enhances Treg functional competency and stability via interfering with the suppressive molecules CTLA-4 and inducible costimulator (ICOS) [102]. Nevertheless, the inhibition of the lipid chaperone fatty acid-binding protein 5 (FABP5), which is important for lipid uptake and intracellular lipid trafficking, impaired OXPHOS and lipid metabolism but promoted Treg functional capacity [97]. The seemed contradictory results require further examinations, such as conducting experiments that eliminate the interference of local metabolism-regulating cytokines, including TGF-β.

Amino acid metabolism and nutrient signals are crucial for Treg functional programming. The role of amino acids, especially arginine and leucine, in driving effector Treg function has been revealed (Fig. 4) [110]. Mechanistically, amino acid signals, interacting with the small G proteins Rag A/B and Rheb 1/2, which are essential for mitochondrial and lysosome fitness and the Treg suppressive gene signature, activate and maintain TCR-induced mTOR activity in Treg cells, thereby licensing effector Treg suppressive capacity [110]. Likewise, L-arginine promoted neonatal Treg function by stimulating IL-10 production, which was accomplished by inducing DNA methylation in the IL-10 promoter region [130]. In addition, synthesized by the enzyme glutamate cysteine ligase (Gclc) with glutamine, glycine, and cysteine as substrates, GSH also enhanced Foxp3 expression and stimulated Treg suppressive capacity, simultaneously impairing the import and synthesis of serine (Fig. 4) [131]. Serine is a nonessential amino acid that actively participates in the one-carbon metabolic network and regulates AA homeostasis and the Teff response [132]. It was shown that suppressing the upregulated serine in Gclc-deficient Treg cells restored Foxp3 expression and its suppression on effector T cells, proving that serine acted as a negative regulator of Treg function [131].

Immunomodulatory metabolites also participate in the modulation of Treg function; as an active derivative of vitamin A, retinoic acid (RA) was demonstrated to play an essential role in maintaining Treg stability and suppressive function under the inflammatory milieu [133, 134]. Nevertheless, this effect can be adverse depending on the dose of RA and environmental conditions, as a recent study has shown that the ablation of intrinsic RA signaling enhanced the suppressive capacity and metabolic fitness of Tregs via stimulating STAT5 and mTORC1 signaling [135].

Collectively, the suppressive function and stability of Tregs are supported by OXPHOS and FAO; in contrast, glycolysis suppresses Treg suppressive capacity. The role of amino acids and nutrient signals in regulating Treg function should not be neglected.

## The altered metabolic landscape of the TME supports TI-Treg proliferation and function through metabolic reprogramming

**Fig. 5:**
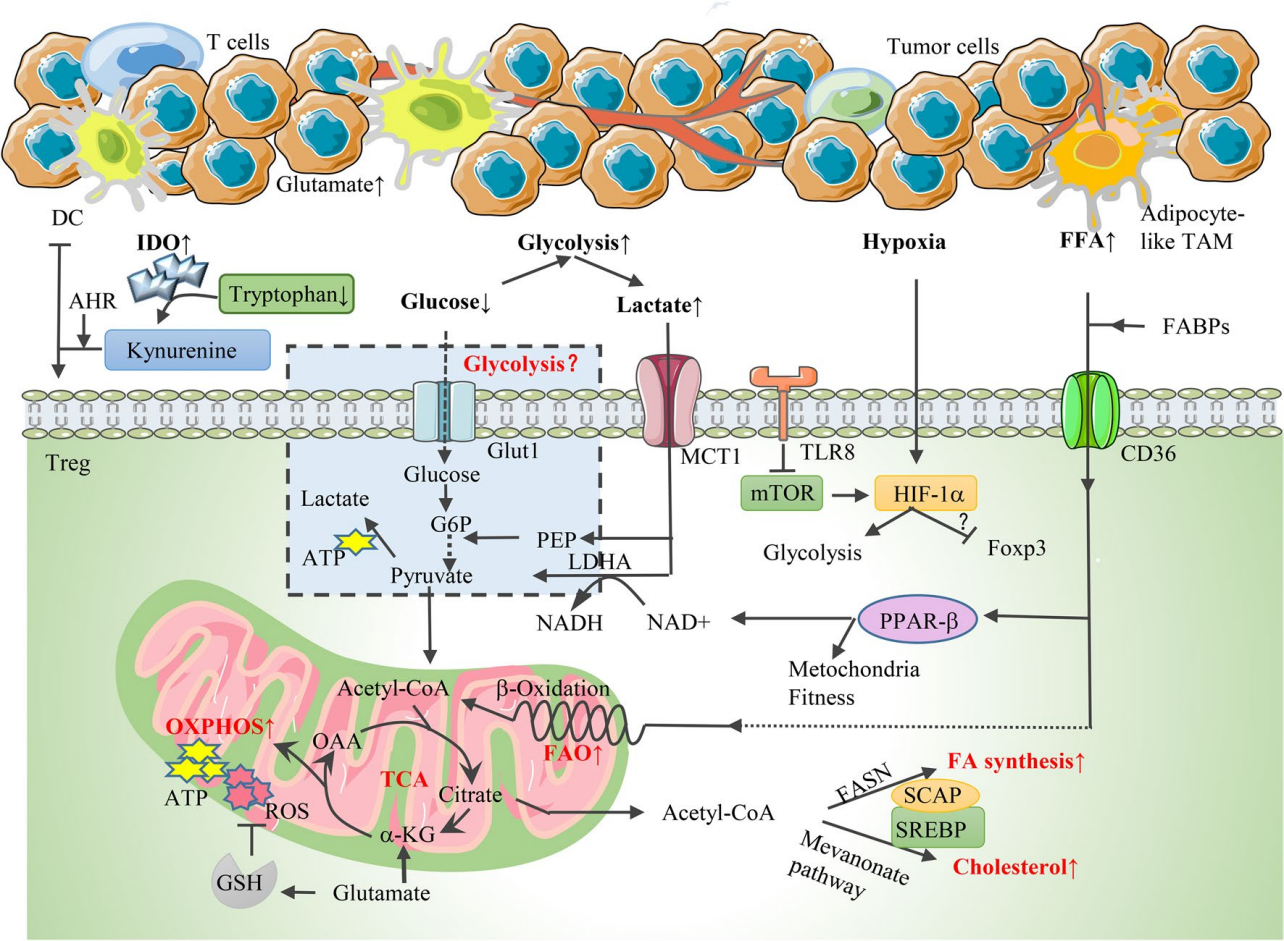
A schematic overview of the influence of the TME on TI-Treg proliferation and function through metabolic reprogramming. **1** The “Warburg effect” characterizes the TME as glucose-deficient and lactate-accumulative. The role of glycolysis in TI-Treg function is controversial, but it is clear that MCT1-induced lactate uptake is increased in Tregs. Lactate is not only converted to pyruvate by LDH to enter the TCA cycle, but is also used for the production of PEP, which serves as an intermediate for glycolysis. **2** FAO and FAS are both active in TI-Tregs. Since FFA availability is upregulated in the TME, the uptake of FA and subsequent FAO are increased with the support of FABPs and CD36. CD36-induced FFA uptake also activates PPAR-β signaling, which promotes mitochondrial fitness and increases the NAD-to-NADH ratio. The mevalonate pathway and FAS are supported by SCAP/SREBP signaling. **3** TI-Tregs show high OXPHO and ROS production, which can be scavenged by GSH. IDO is enriched in the TME, and the IDO-catalyzed tryptophan metabolite kynurenine curbs the infiltration of Teffs by binding to AHR, thus promoting the generation of Tregs by inducing tolerogenic dendritic cells. **4** The hypoxic TME activates the HIF-1α pathway, which promotes glycolysis. The activity of mTOR-HIF-1α is inhibited by TLR8. Until now, the correlation between HIF-1α and Foxp3 expression remains controversial. *MCT1* monocarboxylate transporter 1, *LDH* lactate dehydrogenase, *PEP* phosphoenolpyruvate, *FABP* fatty acid-binding protein, *SREBP* sterol regulatory element-binding protein, *SCAP* SREBP cleavage-activating protein, *IDO* indoleamine 2,3-dioxygenase, *AHR* aryl hydrocarbon receptor, *MDSC* myeloid-derived suppressive cells, *TAM* tumor-associated fibroblasts

Cancer cells are characterized by rapid proliferative rates and high nutrient acquisition [1]. Strikingly, it was discovered by Warburg in the 1950s that cancer cells were reprogrammed to utilize glycolysis rather than oxidative metabolism as a dominant energy resource even under aerobic circumstances to supply increased energetic and biosynthetic demands [136]. This intriguing phenomenon was known as the “Warburg effect” [136]. Consequently, the nutrient-deprived, hypoxic, acidic, metabolite-accumulating tumor microenvironment was formed (Fig. 5), dampening antitumor responses by suppressing the activity of effector and cytotoxic T cells [137]. However, regulatory T cells are abundantly infiltrated in the TME and are resistant to metabolic suppression. Moreover, accumulating evidence suggests that TI-Treg cells flexibly switch to different metabolic programs for adaptation and participate in the formation of an immunosuppressive TME, thus greatly hindering antitumor immunity [138, 139]. Here, we will discuss the characteristics of cancer cell metabolism and their influences on Treg accumulation and suppressive function in the TME, highlighting the difference in the Treg metabolic profiles between the TME and peripheral tissues.

### Glucose deprivation

As there is a lack of glucose availability in the TME, it makes sense that Tregs disfavor glycolysis and switch to other metabolic programs for their survival. This statement has been supported by several studies. For example, in the MC38 colon adenocarcinoma murine model, the level of glucose uptake of intratumoral Tregs remained unchanged compared with splenic counterparts [24]. It was demonstrated that the metabolic adjustment of Tregs in the low-glucose and high-lactate environment was mediated by the key transcription factor Foxp3, which suppressed Myc gene expression and inhibited glycolysis, whereas it promoted OXPHOS [119]. Furthermore, the efficacy of anti-CTLA-4 therapy was enhanced in glycolysis-defective tumors due to the destabilization of Treg suppressive function toward aberrant IFN-γ production [26]. In this study, as tumor cells spare more glucose for TI-Tregs to use, the glucose uptake and glycolysis of Tregs are elevated, contributing to the loss of stability and suppressive function in vitro [26]. Additionally, it was reported that glucose uptake and glycolysis were greatly hampered in TI-Tregs because glucose avidity was correlated with their poorer suppressive function and subsequent instability [25]. Similarly, in AOM-DSS-induced murine colorectal cancer models, enhancing glucose uptake and glycolysis via the suppression of the MondoA-TXNIP axis, the key regulator of glucose metabolism, impaired Treg immunosuppressive function in vitro and subsequently induced the differentiation of Th17-like Treg cells [140]. This Treg subset secreted IL-17A and promoted the progression of colorectal cancer by exhausting CD8^+^ T cells [140].

In contrast, some other studies implied that TI-Tregs engaged in glycolysis and upregulated glucose transporters for their functionality in the TME. In human ovarian cancer, the expression levels of glucose metabolism-related genes and proteins, such as Glut1, Glut3, HIF-1α, and glucose-6-phosphate isomerase, were significantly upregulated compared with those in benign ovarian tumors and healthy controls, as well as CD4^+^ Teffs [141], in agreement with the data from a study based on colorectal cancer and liver cancer [140, 142]. The activation of the Toll-like receptor 8 (TLR8) signal and its ligand can attenuate Treg functionality directly or indirectly [141, 143]. Through inhibiting downstream mTOR-HIF-1α signaling, TLR8 impaired the glucose metabolism of Tregs, leading to the loss of function of intratumoral Tregs and enhancing antitumor immunity (Fig. 5) [141, 143], in line with the result observed by the administration of the glycolysis inhibitors 2-DG and galloflavin [141]. Likewise, the curcumin analog GO-Y030 acted as an inhibitor of the mTOR-ribosomal S6 kinase pathway and attenuated glycolysis in nTregs in vitro, thus restraining the production of the immunosuppressive cytokines IL-10 and TGF-β in Tregs and suppressing their function [144].

The difference may be attributed to the distinct tumor types, and specific experimental settings (e.g., in vivo or in vitro), and further investigations are needed to understand the glucose metabolism features of intratumoral Tregs by adding other cancer types and excluding factors that may interfere with the experimental results, such as the immunosuppressive factors IL-10 and TGF-β in the milieu.

### Enhanced lipid metabolism and mitochondrial fitness

Although whether Tregs utilize glycolysis for survival and function in the TME remains controversial, it is clear that intratumoral Tregs positively engage in lipid metabolism as supplemental metabolic routes. Understanding the role of lipid biosynthesis in regulating Treg bioactivities in the TME has been a hot topic in recent years. It was observed that blockade of FAS has a more profound impact on Treg expansion in vitro than FAO, indicating that FAS may play a dominant role in regulating active Treg proliferation in cancer as well as inflammatory conditions [142]. In this study, Tregs showed enrichments in both genes correlated with glycolytic and lipid synthetic pathways in human liver cancer [142]. Elevated capture and utilization of glucose promoted glycolysis and increased FAS as well as intracellular lipid accumulation of tumor-infiltrating Tregs [142]. In agreement, data from gene enrichment analysis confirmed that lipid metabolism-correlated genes comprised the most enriched gene signature in intratumoral Tregs [24]. It was revealed that sterol regulatory element-binding protein (SREBP), assisted by the activity of SREBP cleavage-activating protein (SCAP), was the dominant mediator of the de novo FAS of Tregs by stimulating fatty acid synthase (FASN) [24]. SCAP/SREBP signaling also modulates the expression of enzymes for mevalonate metabolism, further regulating cholesterol synthesis and protein geranylgeranylation, which influences the survival and suppressive capacity of Tregs in the TME and blocks antitumor immunity (Fig. 5) [24]. In addition, as lipid availability was significantly elevated in the TME due to the increased FA production of tumor cells and accumulation of adipocyte-like tumor-associated fibroblasts (Fig. 5), the involvement of extracellular lipid uptake in regulating intratumoral Treg metabolic and functional fitness should not be neglected. Human gastric cancer cells bearing RHOA gene mutations exhibited increased FAS via the stimulation of the PI3K-AKT-mTOR axis, and the enhanced free fatty acid (FFA) availability in the TME was competitively consumed by Tregs to fuel FAO for recruitment and function [145]. Notably, intratumoral Tregs showed significant upregulation of several FABPs and CD36, which are responsible for the uptake of long-chain FAs and oxidized low-density lipoprotein (Fig. 5) [146, 147]. Genetic deletion of CD36 inhibited Treg-mediated immunological suppression of tumor-infiltrating lymphocytes and suppressed tumor growth without eliciting severe autoimmunity, providing promising prospects for cancer treatment [147]. Mechanistically, CD36-induced FFA uptake activated PPAR-β signaling, which promoted mitochondrial fitness and increased the NAD-to-NADH ratio, thus conferring upon Tregs a preferential survival and functional advantage in the lactate-enriched TME (Fig. 5) [147]. Collectively, lipid metabolism is essential for sustaining intratumoral Treg expansion and suppressive function, and emerging studies reveal the potential of targeting lipid metabolism-related pathways for cancer therapy.

### Oxidative stress and amino acid metabolism

In the low-glucose and high-lactate TME, Tregs are reprogrammed by Foxp3 and preferentially switch to OXPHOS and FAO for metabolic adaptation [119]. Correspondingly, tumor-infiltrating Tregs showed active mitochondrial activity, followed by the excessive production of intracellular ROS, which may disrupt the activity of immune cells [65, 148]. Due to the insufficient antioxidant effect induced by NRF2, intratumoral Tregs are highly sensitive to oxidative stress and exhibit an elevated apoptosis rate due to ROS production [65]. Unexpectedly, Tregs showed increased immunosuppressive effect simultaneously [65]. Mechanistically, via pannexin-1 channels, apoptotic Treg cells released a high amount of ATP, which was then metabolized to adenosine through the ectonucleotidases CD39 and CD73, and signal transduction through adenosine and the A2A receptor antagonize CD8^+^ T-cell-induced antitumor immunity [65]. Other studies supported that human Tregs abundantly expressed antioxidant factors, including thioredoxin-1 and GSH, to abolish ROS-induced cell death [131, 149]. Compared with Tcons, Tregs expressed higher levels of GSH, which was responsible for scavenging ROS to ensure the stable survival of Tregs in the TME (Fig. 5) [131]. In addition to GSH, several studies proved that tumor cells presented significant intracellular glutamate content due to the conversion of glutamine. The increased level of glutamate in the TME promoted Treg infiltration and attenuated antitumor immunity [105, 150, 151]. In addition, it is acknowledged that tryptophan and arginine are the key amino acids regulating the immune response. The metabolic modulation of IDO and its enzymatic pathways in intratumoral Tregs has been widely investigated. IDO was demonstrated to be upregulated in a variety of tumors, promoting cell cycle arrest and energy of Teffs and simultaneously inducing Treg maturation [152–154]. Several IDO1 inhibitors were designed dependent on high-throughput screening, structure-based design, and natural product screening to enhance the efficacy of antitumor treatment [154, 155]. It was confirmed in glioblastoma that the IDO-catalyzed tryptophan metabolite kynurenine curbed the infiltration of Teffs by binding to and stimulating aryl hydrocarbon receptor (AHR), an essential cytoplasmic transcription factor [152]. The stimulation of kynurenine-AHR signaling subsequently promotes the generation of Tregs by inducing tolerogenic DCs (Fig. 5) [152]. In addition, the arginine-catalyzing enzyme arginase 2, which was upregulated in metastatic melanoma compared with healthy skin, was found to enhance Treg suppressive function in vitro as well as Treg recruitment in inflamed tissues in vivo, thus increasing Treg metabolic fitness in the TME and hampering antitumor immunity [156]. Reprogrammed amino acid metabolism represents an essential metabolic node in TI-Tregs, although their specific roles in regulating Treg function in the TME remain unclear and need future investigation.

### Hypoxia

Due to the high consumption of microenvironmental nutrients and the active mitochondrial respiration of tumor cells, there is no sufficient oxygen availability in the microenvironment of solid tumors. It is universally acknowledged that the hypoxic TME induces stimulation of the HIF pathway and strongly influences the transcription of genes related to vascularization, chemoresistance, glycolysis, immune escape, and tumor metastasis [157–160]. A large number of studies have highlighted the involvement of HIF-1α in regulating Treg biological behavior but are still controversial. HIF-1α is responsible for the recruitment and migration of Tregs to the TME by promoting vascular endothelial growth factor A and/or increasing the glycolytic rate of Treg cells [161, 162]. However, the correlation between HIF-1α expression and Treg suppressive function is widely debated. Some studies showed that Tregs lacking HIF-1α restored their ability to suppress the immune response, which was due to the mechanism by which HIF-1α could bind to the promoter region of Foxp3 and cause its degradation under hypoxic conditions, thus destabilizing Foxp3 and causing Treg dysfunction [87]. In contrast, it was proven that HIF-1α was essential for Treg abundance and function under hypoxia by binding to hypoxia response elements upstream of Foxp3 and elevating its transcription [94, 95]. Indeed, although HIF-1α disturbs the transport of pyruvate into mitochondria, the motility and suppressive function of Tregs are sustained due to the subsequently increased glycolysis and FAO [120]. Additionally, enhanced survival was observed in a HIF-1α-ablated murine glioma model, as glycolytic-driven migration of Tregs to the TME was significantly reduced, but their suppressive capacity was maintained via increased OXPHOS [120]. Although these limited studies were insufficient to elucidate the participation of HIF-1α and its complex regulatory networks in regulating TI-Tregs, they significantly facilitated further explorations.

### Accumulation of immunomodulatory metabolites

Excessively produced by proliferating tumor cells, lactic acid is enriched in the TME, where it is usually considered a waste product of glycolysis and exerts an immunosuppressive effect [119, 163, 164]. Tumor-equivalent accumulation of lactic acid is deleterious for conventional T cells and curbs the immune response in vitro, whereas it fails to disturb TI-Treg functionality and represents an alternative fuel for Treg cellular function [119]. Tregs cultivated in a lactate-rich environment appeared to have superior suppressive ability to the control group [144]. The expression levels of Slc16a1 and lactate dehydrogenase (LDHA), which encode lactate transporter, monocarboxylate transporter (MCT1) and LDH, respectively, were significantly upregulated [25]. Intratumoral Tregs appeared to increase lactate uptake via MCT1 [25, 165]. Lactate is converted to pyruvate by LDHA to enter the TCA cycle and fuel mitochondrial respiration, thus sustaining Treg-induced immunosuppression, and this process is supported by LDH and the increased ratio of NAD/NADH [25]. Tregs also use lactate-derived carbon for the production of phosphoenolpyruvate, which then serves as an intermediate for glycolysis and promotes Treg proliferation (Fig. 5) [25]. A recent study demonstrated that lactate absorption in eTregs upregulated the expression of PD-1, thereby strongly influencing Treg suppressive function and the efficacy of PD-1 blockade therapy [165]. Tregs survive in the low-glucose and high-lactate TME, acquiring enhanced proliferative ability and functionality with the support of lactate. In addition to lactic acid, other metabolites, such as D-2-hydroxyglutarate (D-2HG) and RA, also affect Treg survival and function in the TME. Derived from DCs as a metabolite of vitamin A, RA indirectly stimulated TI-Treg activity by supporting the effect of TGF-β and IL-2 on Treg differentiation [166, 167]. Acute myeloid leukemia blasts bearing isocitrate dehydrogenase (IDH) gene mutation released a high level of the oncometabolite D-2HG, which promoted intratumoral Treg expansion and accumulation by impairing HIF-1α stabilization and shifting from glycolysis to OXPHOS for energy demand [168]. In contrast, in lower-grade glioma and secondary GBM, 2-HG accumulation was proven to be negatively correlated with Treg frequency in the TME [169], providing future directions for elucidating the potential mechanisms underlying these observations.

## Targeting Treg metabolic pathways to enhance the efficacy of cancer immunotherapy: therapeutic potential

Recent decades have witnessed massive progress in the field of antitumor immunotherapy. Immune checkpoint inhibitors (ICIs), such as anti-CTLA-4 and anti-PD-1/PD-L1 monoclonal antibodies, which can unleash immunosuppression and reconstitute the antitumor immune response by reactivating dysfunctional or exhausted effector T cells, have achieved remarkable progress in treating patients bearing different types of cancer or in advanced stages [170–176]. However, immunosuppressive tumor-resident Tregs have always been a significant obstacle to ICI treatment, as they can induce drug resistance due to their high proportion in the TME and superior suppressive capacity. Thus, removing TI-Tregs alone or in combination with ICI therapy appears to be attractive for cancer treatment. Strategies targeting Treg-expressed immunosuppressive molecules (e.g., anti-CD25, anti-CTLA-4, GIRT agonist, and anti-OX40) and blocking chemokine/chemokine receptors (e.g., anti-TGFβ, anti-CCR4, and anti-CCR8) have been extensively elucidated in diverse reviews [42, 59, 177]. However, severe autoimmunity often occurs after Treg deletion due to the systemic impairment of their suppressive function. In this context, we will concentrate on the emerging therapeutic potential of disturbing adaptative metabolic pathways of Tregs in the TME (Table 1), hoping to provide new insights into Tregs-targeted therapies.

**Table 1:** Targeting metabolic pathways and their regulators in intratumoral Tregs

Target	Drug	Mechanisms of action	Effect on Tregs
Glycolysis	2-DG, 2-ME, galloflavin, dichloroacetate [89, 141–143]	Inhibit glucose uptake and glycolysis	Inhibit Treg suppression on Teff proliferation
TLR8	Poly-G3, ssRNA40 [141, 143]	Act as TLR8 agonists; inhibit glucose uptake and glycolysis by disrupting the mTOR-HIF-1α axis	Inhibit Treg suppression on Teff
mTOR	Rapamycin, curcumin analog GO-Y030 [144, 178]	Inhibit mTOR signal, glucose uptake, and glycolysis	Inhibit Treg population and immune-suppressive ability
mTORC1	Metformin [179]	Act as a mTORC1 agonist; induce metabolic change from OXPHOS to glycolysis	Inhibit iTreg differentiation, especially terminally differentiated KLRG1^+^CD103^+^Treg cells
PTEN	VO-Ohpic [124]	Inhibit PTEN signal and stimulate PI3K-AKT axis	Cause Treg instability and conversion into ex-Tregs as well as inhibit Treg suppressive function
PI3K	Wortmannin [180]	Inhibit PI3K, TCR, and Treg-specific signals	Selectively inhibit Treg proliferation and function
Pan-PI3K	Copanlisib [183]	Undefined	Decrease Treg cell infiltration
PI3K-β	GSK2636771 [145]	Inhibit PI3K-β signal and interfere with Treg lipid metabolism	Decrease Treg cells
PI3K-δ	Idelalisib (previously CAL-101, GS-1101), PI-3065, parsaclisib [181, 182, 184, 185]	Inhibit PI3K-δ, AKT phosphorylation TCR and Treg-specific signals	Reduce Treg number, differentiation, and function
AKT	Triciribine [180]	Inhibit AKT, TCR, and Treg-specific signals	Selectively inhibit Treg proliferation and function
ACC	TOFA [142]	Inhibit ACC and FAS	Inhibit Treg proliferation
CD36	A monoclonal antibody blocking CD36 [147]	Inhibit FA transport, lipid metabolism as well as mitochondrial fitness and biogenesis	Inhibit intratumoral Treg accumulation and suppression without systemic loss of Treg number and function
CPT1a	Etomoxir [120]	Inhibit CPT1a and FAO	Decrease Treg number
PDH	CPI-613 [178]	Inhibit PDH; block the TCA cycle and mitochondria metabolism	Decrease Tregs and Bregs
IDO1	Secondary sulfonamides (compound 5d), GDC-0919, epacadostat, NLG802 [41, 152, 154, 186]	Inhibit IDO1-catalyzed kynurenine pathway	Inhibit Treg differentiation and suppression
MCT-1	AZD3965 [25]	Inhibit lactate uptake and its support to Treg function	Selectively inhibit intratumoral Tregs without disturbing pTregs
L-lactate	Curcumin analog GO-Y030 [144]	Inhibit L-lactate production by melanoma cells	Inhibit the support of extracellular lactate on Treg function

*2-DG* 2-deoxyglucose; *2-ME* 2-mercaptoethanol; *TLR* Toll-like receptor; *PTEN* phosphatase and tensin homolog; *TCR* T cell receptor; *ACC* acetyl-CoA carboxylase; *TOFA* 5-tetradecyloxy-2-furoic acid; *FAS* fatty acid synthesis; *CPT1* carnitine palmitoyltransferase-1; *PDH* pyruvate dehydrogenase; *IDO* indoleamine 2; 3-dioxygenase; *MCT1* monocarboxylate transporter 1

### Glucose metabolism

The role of glucose metabolism in the regulation of Treg metabolism has always been a controversial issue. The results from different studies vary greatly due to the differences in the proliferative and functional status of Treg cells, experimental settings (e.g., in vivo or in vitro culture systems, human or mouse models), and nutrient and cytokine compositions within the extracellular environment. Some studies proved that the pharmacological inhibition of glycolysis in TI-Tregs through 2-DG, 2-mercaptoethanol, galloflavin, or dichloroacetate significantly attenuated their proliferation and suppression of Teff activity, suggesting that the survival of Tregs in the TME was highly dependent on glucose metabolism [89, 141–143]. Foremost in importance among regulators of glucose metabolism in Tregs is the PI3K-AKT-mTOR axis, which has always been regarded as the predominant therapeutic target for the deletion of Tregs. Emerging evidence shows that TLR8 and its ligand act as suppressors in modulating mTOR-HIF-1α-induced glucose metabolism in intratumoral Tregs, and the TLR8 agonists Poly-G3 and ssRNA40 have been proven to be effective in disrupting Treg-mediated suppression of effector T cells in melanoma and ovarian cancer models [141, 143]. Likewise, the direct inhibition of mTOR activity through rapamycin and the curcumin analog GO-Y030 expectedly reduced the Treg number and suppressive capacity in the TME by restraining glucose uptake and glycolysis [144, 178].

However, as described in the previous section, numerous studies strongly suggested that the PI3K-Akt-mTOR pathway enhanced the glycolytic rate of Treg cells and curbed their differentiation and functional stability. This is supported by evidence that metformin was demonstrated to be a valid mTORC1 agonist inducing iTreg apoptosis at tumor sites [179]. Similarly, the stimulation of the PI3K-AKT axis via VO-Ohpic-induced pharmacological inhibition of PTEN resulted in the generation of loss-of-suppressive-function ex-Tregs, thus creating an inflammatory and immunogenic TME [124]. Notably, PTEN inhibitors synergized with cyclophosphamide or immunotherapy showed high efficacy in elevating antitumor immunity, providing promising prospects for cancer treatment [124].

PI3K and AKT inhibitors, such as wortmannin, copanlisib, GSK2636771, idelalisib, PI-3065, parsaclisib, and triciribine, have been largely documented in experiments or clinical trials investigating their effectiveness in suppressing Treg-induced immunosuppression in different tumors [145, 180–185]. However, these drugs act through mechanisms other than the modulation of glucose metabolism. For example, idelalisib (previously CAL-101, GS-1101), the first PI3K-δ inhibitor for human B cell malignancies, selectively prevented the conversion of CD4^+^ T cells to Tregs and their secretion of immunosuppressive IL-10 by inhibiting AKT phosphorylation and subsequently downregulated the transduction of TCR and expression of Treg-specific signals, including Foxp3, CD25, CTLA4, ICOS, PD-1, and CD39 [181]. Moreover, the PI3K-β inhibitor GSK2636771 decreased FFA production in RHOA-mutant human gastric cancer cells, thus limiting lipid uptake and metabolism in Tregs and suppressing their accumulation and function [145]. In addition, the combination of GSK2636771 and anti-PD-1 achieved superior therapeutic outcomes by relieving PD-1 blockade resistance [145].

### Other metabolic targets

In addition to glucose metabolism, metabolic processes including FAS, FAO, mitochondrial metabolism, amino acid catabolism, and the uptake and conversion of lactate also support Treg metabolic fitness in the TME. Critical regulators of these processes can also serve as potential therapeutic targets for cancer treatment. The administration of 5-tetradecyloxy-2-furoic acid (TOFA), an acetyl-CoA carboxylase (ACC) inhibitor, strongly impaired the FAS cascade in Tregs, showing the expected tumor inhibitory effect [142]. Nevertheless, the reduced tumor growth is predominantly attributed to the direct drug toxicity to cancer cells rather than immune cell reprogramming [142]. In contrast, the effectiveness and safety of a monoclonal antibody blocking CD36, which is responsible for FA uptake and lipid metabolism in Tregs, have been proven in YUMM1.7 melanoma-engrafted murine models [147]. CD36 blockade exhibited promising prospects for cancer immunotherapy or combination therapy with anti-PD-1 through inhibiting TI-Treg accumulation and suppression without systemic loss of Treg number and function, thus preventing severe autoimmunity after treatment [147]. Likewise, interfering with FAO through etomoxir, a CPT1a inhibitor, decreased the Treg ratio to Teff cells, resulting in improved outcomes in brain cancer [120]. Additionally, although mitochondrial metabolism also plays an essential role in modulating TI-Treg activity, investigations on their clinical applications are limited. A study confirmed that CPI-613 restrained the effect of PDH on supporting the entrance of glycolysis to the TCA cycle, thus leading to increased levels of Tregs and Bregs in lysosomal acid lipase-knockout lymph nodes and preventing human lung A549 cancer progression [178]. Further steps are required for uncovering other targets in mitochondrial metabolisms, such as ROS, mitochondrial transcription factors, and mitochondrial respiration chain complexes, for specific Treg ablation in tumors.

Moreover, targeting the IDO-catalyzed kynurenine pathway seems to be attractive for Treg depletion in cancers, as various IDO1 inhibitory drugs have been designed thus far, such as secondary sulfonamides (Compound 5d), GDC-0919, epacadostat, and NLG802, while none of them have been approved for formal clinical treatment of malignancies by the FDA [41, 152, 154, 186]. In addition, the regulation of lactate on TI-Treg metabolism has received much attention in recent years. Blocking lactic acid production through cancer cells and inhibiting lactate transport via MCT-1 blockade seem to be the breakthroughs [41, 144].

## Conclusion and perspective

The increasing understanding of the critical role of Tregs in immune homeostasis, self-tolerance, and immunosuppression has greatly motivated progress in the treatment of autoimmune diseases and cancer. Diverse antitumor strategies that target Tregs alone or in combination with ICIs, chemotherapy, or cancer vaccines are under intense experimental and clinical investigations [180, 186]. Nevertheless, there are still considerable obstacles to the clinical application of Treg cell-targeted therapy, including the lack of strategies to selectively deplete Tregs within the TME without systemic disruption of Treg function, as well as how to minimize the impact on other T cells, such as Teffs. Since cellular metabolic reprogramming is considered a hallmark of cancer, manipulation of cellular metabolism on the viability and function of both cancer cells and immune cells has been extensively explored [1]. In this review, we discuss the common and distinct metabolic profiles of Tregs in peripheral tissues and the nutrient-deprived, hypoxic, lactate-enriched TME. We focus on the essential roles of metabolic programs such as glycolysis, OXPHOS, FAS, FAO, and AA metabolism in modulating Treg proliferation, migration, and function during normal physiology and pathologies including inflammation, autoimmunity, and cancer progression. Moreover, the involvement of critical metabolic regulators, such as the PI3K-AKT-mTOR axis, AMPK, PTEN, HIF-1α, lactate, ROS, CD36, PPARγ, IDO, glutamate, and glutamine, which orchestrate Treg metabolism through different mechanisms, is highlighted. Indeed, Treg cellular metabolism, especially glucose metabolism, is highly heterogeneous and varies significantly in different proliferative and functional statuses of Treg cells, experimental settings (e.g., in vivo or in vitro culture systems, human or mouse models), and nutrient/cytokine compositions within the extracellular environment. Explaining the underlying mechanisms of these heterogeneities in Treg biology and determining how to target Tregs solely within the TME to avoid severe autoimmunity caused by systemic loss of immune tolerance are urgent matters. Future studies will focus on finding more efficient targets for selective Treg depletion in the TME and their combinations with other therapies.

## Data Availability

Not applicable.

## Abbreviations

AA: Amino acid
ACC: Acetyl-CoA carboxylase
AHR: Aryl hydrocarbon receptor
AMPK: AMP-activated protein kinase
CCR: Chemokine (C–C motif) receptor
CNS2: Conserved noncoding sequences 2
CTLA-4: Cytotoxic T lymphocyte antigen 4
CPT1: Carnitine palmitoyltransferase-1
CXCR: C–X–C chemokine receptor
2-DG: 2-Deoxyglucose
D-2HG: D-2-hydroxyglutarate
FAO: Fatty acid oxidation
FABP: Fatty acid-binding protein
Foxp3: Forkhead box protein P3
GSH: Glutathione
Glut1: Glucose transporter 1
GCK: Glucokinase
Gclc: Glutamate cysteine ligase
GBM: Glioblastoma
HIF-1α: Hypoxia-inducible factor 1α
3-HAA: 3-Hydroxyanthranillic acid
IDO: Indoleamine 2,3-dioxygenase
IDH: Isocitrate dehydrogenase
ICI: Immune checkpoint inhibitor
LAG-3: Lymphocyte activation gene 3
LKB1: Liver kinase B1
LDHA: Lactate dehydrogenase
MDSC: Myeloid-derived suppressive cell
mTOR: Mammalian target of rapamycin
MCT1: Monocarboxylate transporter
NRF2: Nuclear factor erythroid 2-related factor 2
OXPHOS: Oxidative phosphorylation
pTreg: Peripheral Tregs
PD-1: Programmed cell death protein-1
PPAR: Proliferator-activated receptor
PI3K: Phosphoinositide 3-kinase
PTEN: Phosphatase and tensin homolog
PDHK1: Pyruvate dehydrogenase kinase 1
PEP: Phosphoenolpyruvate
Treg: Regulatory T cell
ROS: Reactive oxygen species
STAT5: Signal transducer and activator of transcription 5
SREBP: Sterol regulatory element-binding protein
SCAP: SREBP cleavage-activating protein
RA: Retinoic acid
TME: Tumor microenvironment
TIL: Tumor-infiltrating lymphocyte
Tcon: Conventional T cell
TCR: T cell receptor
TI-Treg: Tumor-infiltrating Treg
Teff: Effector T cell
TCA: Tricarboxylic acid cycle
TLR: Toll-like receptor
Tfam: Mitochondrial transcription factor A
TOFA: 5-Tetradecyloxy-2-furoic acid
VAT: Visceral adipose tissue
